# Dermatophyte infection: from fungal pathogenicity to host immune responses

**DOI:** 10.3389/fimmu.2023.1285887

**Published:** 2023-11-02

**Authors:** Ruixin Deng, Xiaowen Wang, Ruoyu Li

**Affiliations:** ^1^ Department of Dermatology and Venerology, Peking University First Hospital, Beijing, China; ^2^ Research Center for Medical Mycology, Peking University, Beijing, China; ^3^ National Clinical Research Center for Skin and Immune Diseases, Beijing, China; ^4^ Beijing Key Laboratory of Molecular Diagnosis on Dermatoses, Beijing, China

**Keywords:** dermatophyte, protease, innate immune response, pattern recognition receptors, caspase-associated recruitment domain 9, type 1 immune response, type 17 immune response

## Abstract

Dermatophytosis is a common superficial infection caused by dermatophytes, a group of pathogenic keratinophilic fungi. Apart from invasion against skin barrier, host immune responses to dermatophytes could also lead to pathologic inflammation and tissue damage to some extent. Therefore, it is of great help to understand the pathogenesis of dermatophytes, including fungal virulence factors and anti-pathogen immune responses. This review aims to summarize the recent advances in host-fungal interactions, focusing on the mechanisms of anti-fungal immunity and the relationship between immune deficiency and chronic dermatophytosis, in order to facilitate novel diagnostic and therapeutic approaches to improve the outcomes of these patients.

## 1 Introduction

Dermatophytes are the most common pathogenic filamentous fungi, with an infection rate of as high as 20%-25% worldwide (1). Dermatophytes usually infect the nails, skin, and hairs (2, 3), causing multiple superficial dermatophytoses, such as tinea capitis, onychomycosis, tinea corporis, and tinea pedis (4). Unfrequently, dermatophytes may also invade the dermal tissue and even deep organs, particularly in immunocompromised patients with congenital or acquired immunodeficiency (5), and these infections can progress to life-threatening conditions if appropriate treatment is not provided (6).

Dermatophytes can be categorized into three types based on host preferences and ecological niches (7): anthropophilic dermatophytes are mainly transmitted from person to person and usually result in chronic infections with moderate clinical symptoms; zoophilic dermatophytes prefer selective animal hosts but can normally infect other species, including humans, often causing inflammatory skin infections; and geophilic dermatophytes survive on keratinized waste present in the soil and are rarely pathogenic but can produce more severe inflammation than anthropophilic species (8). *Trichophyton rubrum* is among the most frequently detected species globally and is responsible for 50%-90% of dermatophytoses (9–13). However, a sudden shift in the most prevalent pathogen from *T. rubrum* to *T. mentagrophytes* complex in India has been observed in recent years, which might result from advances in molecular identification (14). Other important species include *Microsporum canis*, *Epidermophyton floccosum*, and *T. tonsurans* (**Table 1**) (22, 30, 31). In addition, a new emerging drug-resistant dermatophyte, *T. indotineae*, has caused a concurrent overwhelming circumstantial increase in reports on recalcitrance and drug resistance in India (32–34). This strain has also been isolated from several European, American, and Asian countries and has become a public health issue due to the number of individuals affected and the misery it causes (16, 35–42).

**Table 1:** Primary species of dermatophytes and their typical characteristics.

Ecological niche	Pathogen	Clinical picture	Epidemiology	Ref
Anthropophilic	*T. rubrum*	Tinea pedis Tinea corporis Onychomycosis	Most common species worldwide	(10, 15)
	*T. interdigitale*	Tinea pedis Tinea cruris Onychomycosis	Distributed worldwide	(16)
	*T. tonsurans*	Tinea corporis Tinea capitis	Distributed worldwide	(17)
	*T. violaceum*	Tinea pedis Onychomycosis	Most important species in Africa	(18)
	*M. audouinii*	Tinea capitis	Distributed worldwide	(19, 20)
	*E. floccosum*	Tinea pedis	Distributed worldwide	(21)
Zoophilic	*T. mentagrophytes*	Tinea corporis Tinea capitis Tinea cruris	Second most common species worldwide	(9, 22–25)
	*M. canis*	Tinea capitis	Leading agent of tinea capitis in most parts of Europe and Asia	(26, 27)
	*T. benhamiae*	Tinea corporis Tinea capitis Tinea cruris	Distributed mainly in Japan, Europe, and the United States	(28)
Geophilic	*Nannizzia gypsea*	Tinea corporis Tinea capitis	Rarely infects humans	(29)

In recent years, the prevalence of dermatophytosis has continuously increased, especially in tropical or subtropical countries such as India (21, 43). Several risk factors may further increase the risk of dermatophyte infection, including type 2 diabetes, lack of physical activities, vascular disease, anemia, immunosuppression due to leukemia, organ transplant, acquired immunodeficiency syndrome (AIDS), and the use of immunosuppressants (44, 45). The elevated incidence of dermatophyte infections, especially chronic and recurrent dermatophytosis, has a large impact on patients’ quality of life and often requires extended treatments, causing psychological and economic burden (46–51). To provide a comprehensive understanding of the mechanism of dermatophytosis, this review summarizes recent findings on the pathogenesis of and the host’s immune responses to dermatophyte infections.

## 2 Virulence factors related to the pathogenicity of dermatophytes

**Figure 1:**
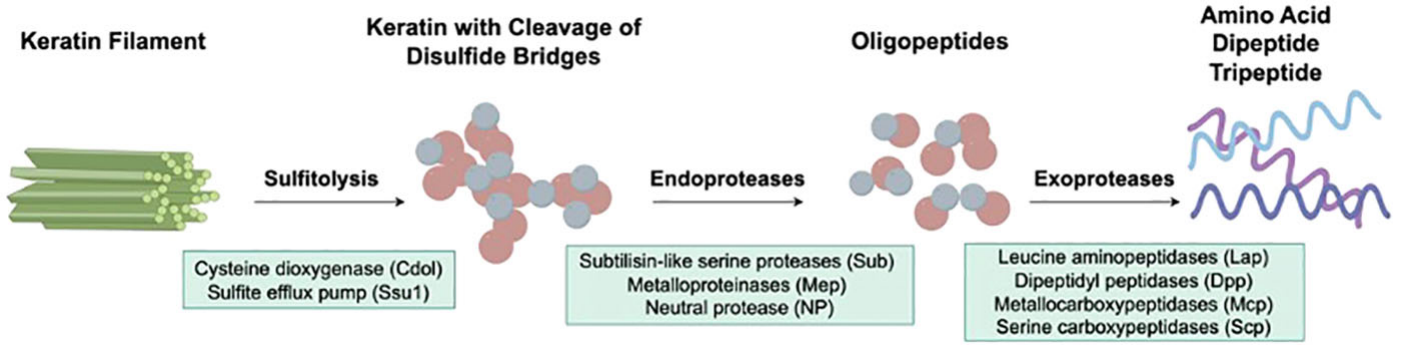
Virulence factors of dermatophytes involved in the keratinolysis process. The initial stage of native keratin biodegradation is sulfitolysis, the key enzymes of which are cysteine dioxygenase (Cdol) and sulfite efflux pump (Ssu1). Then, endoproteases degrade keratin to release free peptides, which are further cleaved into amino acids by exoproteases.

When dermatophytes infect human skin, the first obstacles they need to overcome include physical, chemical, and morphological barriers of the skin. Abnormalities in the stratum corneum, such as macerations and occlusions, may promote fungal infection (52). Once the pathogen crosses the skin barriers mentioned above, colonization begins, and various other processes occur, including adhesion, germination, and invasion. The first stage of infection by arthroconidia includes adherence to the host epidermis via special fungal surface proteins (53). For example, *T. rubrum* binds epithelial cells through carbohydrate-specific adhesins on the microconidial surface, while *T. mentagrophytes* protrudes fibrillar projections when it requires adherence capabilities (54). In the next stage, arthroconidia identify a favorable environment and initiate metabolic reactivation and growth as hyphae (55). During the last stage, the epidermal cornified layer is invaded by the germinating tubes produced by hyphae that secrete various keratinases to digest keratin into smaller peptides and amino acids (56, 57), which are absorbed by dermatophytes as nutrients for growth and reproduction. The initial stage of native keratin biodegradation is sulfitolysis, during which the extensive keratin disulfide bridges are hydrolyzed (58, 59). Afterward, keratin can be further hydrolyzed by various proteases secreted by dermatophytes (60, 61). Endoproteases degrade keratin to release free peptides, on which exoproteases act to further decompose the peptides into smaller peptides and amino acids (62, 63) (**Figure 1**).

### 2.1 Virulence factors involved in the sulfitolysis process

Dermatophytes secrete a variety of proteases, most of which cannot directly hydrolyze natural keratins. Disulfide bonds within keratin molecules can cross-link within and between peptide chains, forming a network structure that is 3-dimensional, making natural keratin difficult to decompose (64). Therefore, cleavage of disulfide bridges must be the first step in keratinolysis. The key enzymes involved in the sulfitolysis process are cysteine dioxygenase (Cdol) and sulfite efflux pump (Ssu1) (65). Kunert and Truper (66) first reported the mechanisms of cysteine metabolism by *N. gypseum* hyphae in 1986: Cdol catalyzes the oxidation of cysteine to cysteine sulfinic acid, which is eventually metabolized to produce sulfites, sulfates and taurine; sulfites can be excreted by Ssu1. Subsequently, researchers found that L-cystine could induce the expression and activation of Cdol in *T. mentagrophytes*, and they successfully isolated recombinant Cdol (67, 68). Moreover, expression of the *CDO1* gene in *T. schoenleinii* was significantly upregulated after coculture with keratin (69). In addition, *CDO1* and *SSU1* knockout strains of *T. benhamiae* showed obvious growth defects when cocultured with human hair and nails, suggesting that Cdo1 and Ssu1 are important virulence factors of dermatophytes (56).

### 2.2 Proteases of the main species of dermatophytes

Subtilisin-like serine protease 3 (Sub3) is the first keratinase found in *M. canis* (70, 71) and is essential for adhesion to the epidermis (72) (**Figure 1**). Subsequently, Brouta et al. (73, 74) revealed the expression of metalloproteinases (Mep) in *M. canis in vitro* and *in vivo* and discovered that metalloproteinases could mediate humoral and cellular immune responses (75). Subsequently, the virulence activities of Mep and Sub have been studied in various dermatophyte species. Mep4, Mep5 and Sub6 proved to be the predominant virulence factors of *T. mentagrophytes* (76, 77), while *SUB6*, *SUB7*, *MEP1*, *MEP2* and *MEP5* expression in *T. tonsurans* was associated with invasion (78). Leng et al. (79) demonstrated that Sub3, Sub4 and Mep4 were required for *T. rubrum* to invade skin. The expression of *SUB1*, *SUB6-7*, and *MEP3* was upregulated significantly after culturing in a medium containing nail chips, illustrating that these genes could contribute to the pathogenicity of *T. rubrum* as well (80, 81). Recently, Baumbach et al. (82) observed *SUB3* and *SUB6* expression in *T. benhamiae in vitro* and *in vivo* using immunofluorescence methods, indicating that Sub3 and Sub6 might play a primary role in the adhesion and invasion of *T. benhamiae*. Apart from endoproteases, exoproteases also draw tremendous attention in research on the pathogenicity of dermatophytes (**Figure 1**). In 2005, Monod et al. (83) identified leucine aminopeptidase (Lap) 1 and Lap2 and dipeptidyl peptidase (Dpp) IV and DppV produced by coculturing *T. rubrum* with keratin, which were also found in *T. violaceum* and *T. benhamiae* (62, 84–86). In addition, the expression level of family of serine hydrolases 1 (FSH1) in *M. canis* cocultured with infant scalp was significantly increased (87), and *FSH1* knock-out significantly decreased *M. canis* virulence (88), proving FSH1 as a potential virulence factor of *M. canis*.

### 2.3 Adaptation to skin pH changes

The pH of healthy skin and nails is slightly acidic; however, the amino acid metabolism during keratin breakdown causes an alkaline shift (89). Accordingly, the dermatophyte keratinases released during the early infection stages exhibit optimal activity at a slightly acidic pH, and other keratinases found in the later phases during keratin breakdown have maximal activity at higher pH values (90), which demonstrates that pathogenic fungi can sense and respond to the environmental pH. This adaptive response relies on the conserved *PacC/Pal* signal transduction pathway, which includes active PacC protein as a pH signaling transcription regulator (91, 92). PacC is essential for dermatophytes to grow on human tissues, as *PacC* gene disruption reduces keratinolytic protease secretion and the ability of mutant strains to invade the stratum corneum (93). **Table 2** summarizes the main virulence factors of dermatophytes.

**Table 2:** Primary virulence factors of dermatophytes.

Virulence factors	Description	Ref
Endoproteases	Subtilisin-like serine proteases (Sub1-12), metalloproteinases (Mep1-5), and neutral protease (NP-I, NP-II)	(58, 94, 95)
Exoproteases	Leucine aminopeptidases (Lap1-2), dipeptidyl peptidases (DppIV, DppV), metallocarboxypeptidases (McpA, McpB), and serine carboxypeptidases (ScpA, ScpB)	(62, 94)
Other proteases	Lipases, glucanases, elastases, gelatinases, phosphatases, and DNases	(62, 94, 96)
Enzymes involved in secondary metabolite production	Polyketide synthase and nonribosomal peptide synthetase	(62, 97)
Enzymes involved in sulfitolysis	Cysteine dioxygenase (Cdol) and sulfite efflux pump (Ssu1)	(56)
Hydrophobins	Avoidance of immune recognition by neutrophils	(98)
Heat shock proteins (Hsps)	Adaptation to human temperature, transformation of dimorphic fungus, keratin degradation and drug resistance	(99)
*PacC* gene	Involved in the regulation of protease expression to adapt to skin pH changes	(93)
LysM domain	Binding to skin glycoproteins; involved in immune evasion	(100)
*ZafA* gene	Growth, reproduction, and zinc absorption	(101, 102)

### 2.4 Mechanisms of antifungal resistance

There has been increasing concern about antifungal resistance during the last decade, especially after the emergence of *T. indotineae*, which can cause chronic or recurrent widespread superficial infections (103, 104). The frequency of terbinafine resistance in *T. indotineae* isolates is approximately 75% in India and more than 15% in other countries (33, 105). In addition, clinical failure of *T. indotineae* infection to azole treatments has also been reported globally (16, 35, 106, 107). Typically, antifungal resistance is acquired due to changes that directly or indirectly affect the drug–target interaction, including genetic changes to the target binding site (108), elevated drug efflux activity for intracellular drugs (103), or inhibition of prodrug activation (109). In terms of *T. indotineae*, terbinafine resistance has been associated to single nucleotide polymorphisms in the gene encoding the terbinafine binding protein squalene epoxidase (SQLE). In terms of *T. indotineae*, terbinafine resistance has been associated with single nucleotide polymorphisms in the gene encoding the terbinafine binding protein squalene epoxidase (SQLE). The Leu393Phe, Leu393Ser, and Phe397Leu substitutions in the enzyme have been the most frequently reported from resistant isolates (36). In addition, the overexpression of the ATP-binding cassette transporter (ABC) family gene MDR3 and the amplification of the C14-α-demethylase-encoding gene CYP51B are associated with azole resistance (40, 110, 111).

## 3 Host immune responses to dermatophytes

Apart from the direct damage caused by fungal virulence factors, host immune responses to dermatophyte infection also result in inflammatory damage to skin. Host immune responses, including those of the adaptive and innate immune systems, are determined by the interaction of pathogen-associated molecular patterns (PAMPs) and damage-associated molecular patterns (DAMPs) with pattern recognition receptors (PRRs) (1).

### 3.1 Innate immune responses

In shaping immune responses, dermatophytes can be detected by nonimmune and immune mediators via their cell wall components, secreted extracellular molecules, or intracellular content by multiple PRRs, which, when bound, cause transduction of intracellular signals that stimulate phagocyte lysis, cytokine and chemokine secretion, fungal phagocytosis, respiratory burst, etc. (112, 113). Usually, PRRs are classified according to their composition and activities: C-type lectin receptors (CLRs), retinoic acid inducible gene (RIG)-like receptors (RLRs), Toll-like receptors (TLRs), and nucleotide-binding and oligomerization domain (NOD)-like receptors (NLRs) (114, 115). Dermatophytes interact with the TLR, CLR, and NLR signaling pathways, which regulate host antifungal immunity.

**Figure 2:**
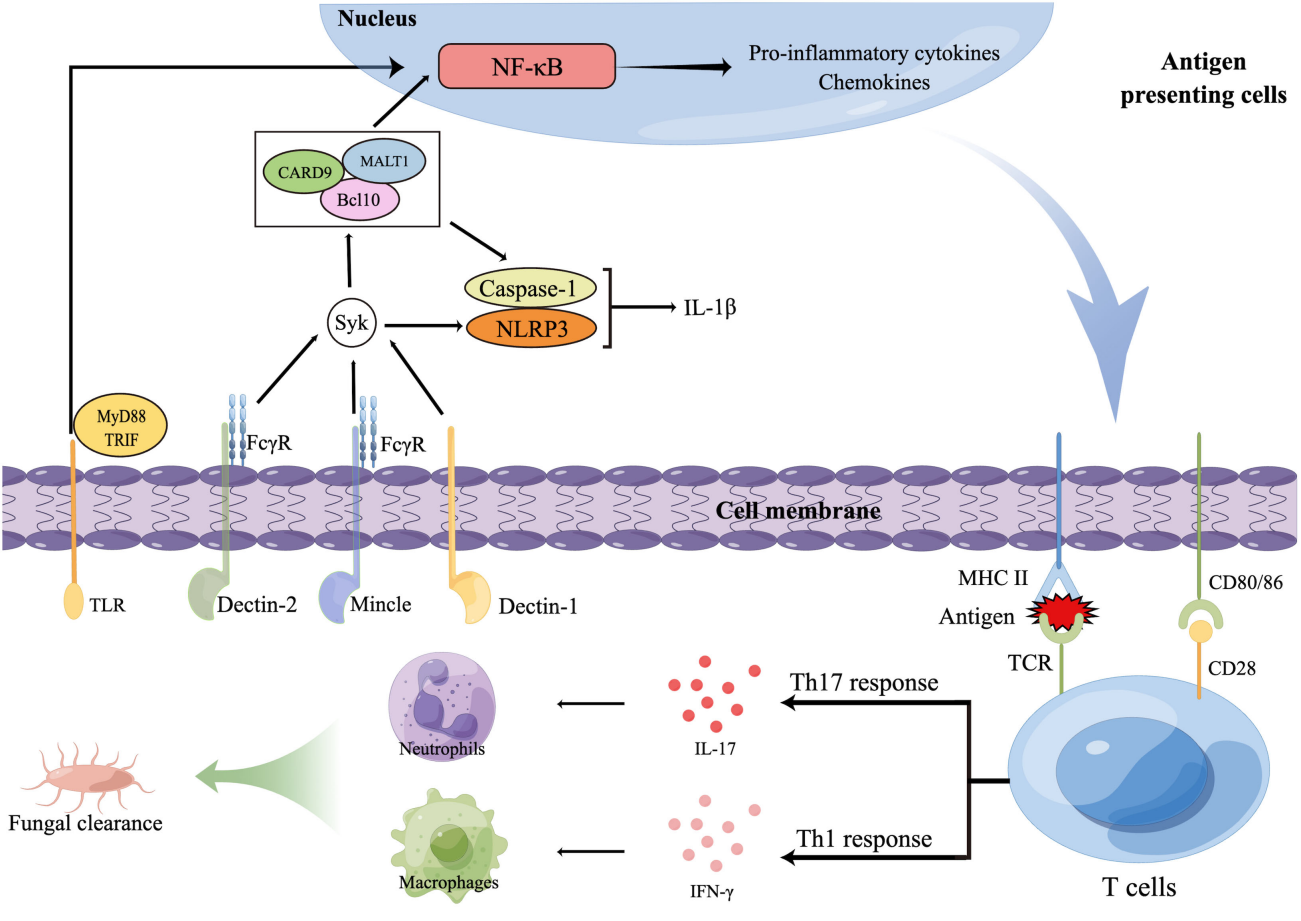
Innate and adaptive immune responses against dermatophytes. Several pattern recognition receptors (PRRs) on antigen presenting cells (APCs), including C-type lectin receptors (CLR) and TLR, are able to recognize dermatophytes, resulting in the release of transcription factors to nucleus that will regulate expression of inflammatory cytokinesThe foreign antigens APCs present and pro-inflammatory cytokines APCs secrete promote the polarization of T cells. T helper 17 (Th17) cells and other IL-17-secreting T cells (not shown) produce IL-17, promoting the production of neutrophil-recruiting chemokines. Interferon γ induced by Th1 cells activates macrophages. Then, macrophages and neutrophils together phagocytose and kill dermatophytes. Bcl10, B-cell lymphoma/leukemia 10; CARD9, Caspase-associated recruitment domain 9; FcγR, Fc receptor γ-chain; IFN-γ, Interferon γ; IL, Interleukin; MALT1, Mucosa-associated lymphoid tissue lymphoma translocation protein 1; MHC, Major histocompatibility complex; MyD88, Myeloid differentiation primary response 88; NF-κB, Nuclear factor κB; NLRP3, NOD-like receptor thermal protein domain-associated protein 3; TCR, T-cell receptor; Th cells, T helper cells; TLRs, Toll-like receptors; TRIF, Toll IL-1 receptor domain (TIR)-comprising adaptor inducer interferon-β.

CLRs, including dectins, Mincle, and mannose receptor (MR), recognize glycans, glycolipids, and glycoproteins contained in fungal cell walls that are not found in mammals (113, 114). Expressed on myeloid cells (neutrophils, dendritic cells [DCs], monocytes, and macrophages), keratinocytes, and human B-cell and T-cell subsets (116, 117), dectin-1 recognizes many pathogenic fungi by β-1,3-glucans present on their cell wall (112) and is thus one of the most important receptors in antifungal immune activities. The immunoreceptor tyrosine-based activation motif (ITAM)-comprising cytoplasmic domain is involved in dectin-1 signaling and is phosphorylated to recruit Syk kinase via a Src family kinase, activating mucosa-associated lymphoid tissue lymphoma translocation protein 1 (MALT1), caspase-associated recruitment domain 9 (CARD9), and a molecular scaffold composed of B-cell lymphoma/leukemia 10 (Bcl10) (118). This activation subsequently activates the nuclear factor κB (NF-κB) pathway, the canonical (NOD-like receptor thermal protein domain-associated protein 3 [NLRP3]/caspase 1) and noncanonical (MALT1/caspase 8) inflammasomes, and the Raf-1 kinase pathway (119) (**Figure 2**).

Dectin-2 and Mincle belong to the dectin-2 cluster, which are predominantly expressed on myeloid cells and can identify O-linked mannoproteins and α-mannans from multiple pathogenic fungi (113). These receptors not only interact with the ITAM-comprising Fc receptor γ-chain (FcγR) to stimulate intracellular signaling through the Syk-CARD9 pathway (120) but also antagonize or synergize with other CLRs, such as TLRs, dectin-1, and inflammasomes (121).

Previous reports have described the function of dectin-1 and dectin-2 in protecting against dermatophyte infection. Both of these proteins can identify and bind to *T. rubrum* and *M. audouinii*, mediating innate immune responses (122). In addition, dectin-1 expression was markedly upregulated in *T. rubrum* cocultured with keratinocytes (123). Defects in these receptors severely impair the production of interferon γ (IFN-γ), tumor necrosis factor α (TNF-α), interleukin-1β (IL-1β), IL-10, and IL-6, culminating in deep dermatophytosis (124–126). These studies illustrated the major role of dectins in antidermatophyte immunity.

As a type-I transmembrane protein mainly expressed by DCs and macrophages, MR is responsible for L-fucose, D-mannose, or N-acetyl glucosamine recognition (113); however, its function in immunity to fungal pathogens seems controversial. MR activation led to Grb2 recruitment followed by Rac/Pak/Cdc-42 signaling cascade activation, which in turn limited phospho-inositide-3 kinase (PI3K) activity and phagosome-lysosome fusion (127). Nevertheless, blockade of MR using soluble antibodies inhibited macrophages from phagocytosing *T. rubrum* spores *in vitro* (128). Therefore, further investigations are required to understand the redundant roles of MR in the innate immune response.

TLRs are type-I integral membrane glycoproteins that can also recognize fungal PAMPs; however, the primary structures of fungal ligands are still only partially resolved to date. TLRs can hetero- or homodimerize with CLRs in the presence of ligands and then mediate intracellular signal transduction by Toll IL-1 receptor domain (TIR)-comprising adaptor inducer interferon-β (TRIF) and myeloid differentiation primary response 88 (MyD88), thereby initiating inflammatory responses.

TLR2 and TLR4 are representative TLRs in the recognition of dermatophytes. Upon coculture with dermatophytes, TLR2 and TLR4 expression in keratinocytes, neutrophils, myeloid cells, and fibroblasts was significantly increased (123, 129, 130). Similar results were obtained in patients with dermatophytosis (131). Interestingly, reduced expression of TLR4 in patients with disseminated dermatophytosis was found compared to that in healthy controls, indicating that the lack of TLR4 might contribute to the lack of infection resolution and the resulting chronic state of dermatophytosis (132). TLR2 blockade by neutralizing antibodies disrupts monocyte fungicidal activity against *T. rubrum* and monocyte TNF-α secretion, suggesting the importance of and requirement for TLR2 for effective conidium phagocytosis, and the absence of TLR2 in human monocytes may disrupt the successful inflammatory response (133). However, another study showed that TLR2-deficient and wild-type (WT) mice exhibited similar control of deep dermatophyte infection; nevertheless, the TLR2-deficient mice exhibited a notable elevation in IFN-γ, IL-10, and IL-17 production and an increased percentage of splenic regulatory T (Treg) cells (134). Therefore, how TLR2 exerts its immune activities during dermatophyte infection is still not completely clear, and more investigations are needed to elucidate its role in protection against dermatophytosis.

Similar to TLRs, NLRs are also associated with antifungal immunity primarily via recognition of other PRRs. NLRP3 is the most important NLR for antifungal immunity, and it is a part of the inflammasome, characterized by subsequent IL-18 and IL-1β generation owing to caspase-1 activation. Previous research has demonstrated the critical role of the NLRP3 inflammasome in host innate immunity against *M. canis* infection since *M. canis*-stimulated IL-1β secretion relies on NLRP3, whereas dectin-1, Syk, and CARD9 are linked with IL-1β secretion via pro-IL-1β transcription modulation (135). In addition, bone marrow-derived macrophages generate IL-1β in response to *T. rubrum* conidia in a caspase-1 and NLRP3-dependent manner, exerting protection against *T. rubrum* infection (136). A similar phenomenon was observed in monocytes during *T. schoenleinii* infection (137). *In vivo* experiments also showed that *T. benhamiae*, *Arthroderma vanbreuseghemii* and *M. canis* could promote IL-1β production in epithelial cells through the NLRP3-caspase1 pathway (138), which played a protective role against dermatophyte infection. Taken together, these results suggest that manipulating NLRP3 signaling can be a novel approach for the control of dermatophytosis.

**Figure 3:**
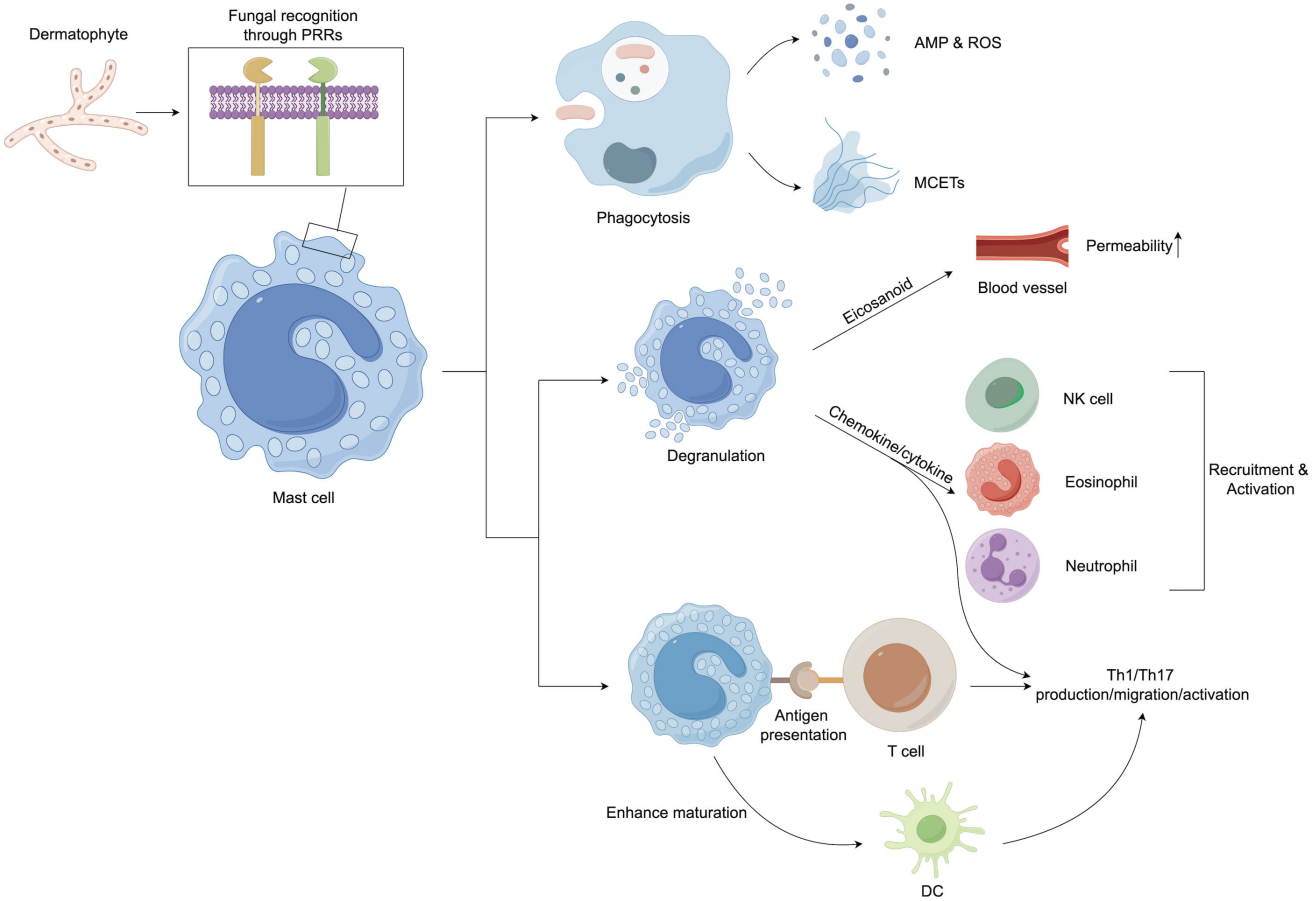
Mast cells play an important role in host defense against dermatophytes. After recognition of the pathogen, mast cells initiate both direct and indirect host defenses. They can kill pathogenic microorganisms by phagocytosis and the production of ROS, AMP, and extracellular traps. In addition, granulation mast cells can release several proinflammatory mediators, including histamine, chemokines, and cytokines, which can upregulate the innate immune response. Mast cell products have also been implicated in the regulation of adaptive immune responses, especially the promotion of Th17 reactions. In addition, mast cells can enhance the maturation of dendritic cells (DCs), priming DCs to facilitate Th17 and Th1 polarization. AMP, Antimicrobial peptide; DCs, Dendritic cells; MCETs, Extracellular traps of mast cells; NK cells, Natural killer cells; PRRs, Pattern recognition receptors; ROS, Reactive oxygen species; Th cells, T helper cells.

Mast cells (MCs) also largely contribute to the immune responses in dermatophytosis infections as they are the first cells to encounter pathogens along with the other innate immune cells (**Figure 3**). MCs can participate in the direct killing of organisms by phagocytosis and the production of reactive oxygen species (ROS) and antimicrobial peptides (AMPs) (139). Another antifungal mechanism of MCs is the formation of extracellular traps composed of DNA, histones, and granule proteins (140). Mast cells can modulate host innate immune responses by secreting eicosanoid metabolites, chemokines, and cytokines (141). The release of histamine and other vasoactive mediators increases vascular permeability and local blood flow to increase the clearance of fungus (139). Chemotactic factors can enhance the recruitment of multiple inflammatory cells, including eosinophils (eotaxin), natural killer (NK) cells (IL-8), and neutrophils (IL-8 and TNF-α) (142).

### 3.2 Adaptive immune responses

Adaptive and innate immunity are inextricably linked, and a successful adaptive immune response represents a cooperative effort that requires stimulation of antigen-presenting cells (APCs), tissue resident cells, and antigen-specific B and T cells (143). The T cell-mediated adaptive immune response is essential in antifungal defense (144, 145). PRR activation initiates a signal cascade that stimulates the MAPK and NF-kB pathways, which in turn induce naive T-cell transformation into T helper (Th) cells (146). Differentiation of Th cells is critical for antifungal immunity, as these cells generate proinflammatory cytokines, such as IL-17 and IFN-γ, which promote the killing of fungi by recruiting and activating phagocytes (146) (**Figure 2**).

The IL-17-mediated (type 17 or Th17) response was reported to be a critical pathway for host defense against fungal invasion (145). The IL-17 family consists of 6 six members (IL-17A to IL-17F) that exert their biological activities via interaction with IL-17 receptors (IL-17RA to IL-17RE). The most prevalent of these members are IL-17A and IL-17F, which exert their biological functions by interacting with IL-17RA and IL-17RC (147). In the skin, myeloid cell PRRs recognize fungi, fibroblasts and keratinocytes and stimulate IL-23, IL-1β, IL-6, and IL-21 synthesis (129, 148, 149); these cytokines interact with their lymphocytic receptors, alternatively inducing the phosphorylation and thus activation of cellular signal transducer and transcription 3 (STAT3), activation of retinoic-acid-receptor-related orphan nuclear receptor gamma (RORγt) transcription factor activity and subsequently stimulating type 17 cytokines (IL-22 and IL-17) or Th17 lineage production (150). Several studies have found that MCs may play an essential role in the functions of Th17 lymphocytes (**Figure 3**). MC-derived TNF is required to develop IL-17-secreting Th17 cells in a murine model (151). MCs can also induce Th17 differentiation in eosinophil-deficient mice, leading to neutrophil-dominant inflammation (152). In addition, MC-secreting chemokines might participate in Th17 infiltration (153). Interestingly, Th17 cells express the functional histamine H4 receptor; thus, MC-derived histamine may affect Th17 activity (154). Indeed, stimulation with histamine or an H4 receptor agonist increases the production of IL-17 by human Th17 cells (154). IL-17 can stimulate the generation of various cytokines (such as TNF and IL-6), vascular endothelial growth factor (VEGF), and chemokines (CXCL1 and CXCL8), increase AMP expression, and promote keratinocyte proliferation (145), which may essentially lead to the clearance of cutaneous fungal infection.

Mounting evidence has illustrated the potent antifungal immunity of AMPs induced by IL-17, which mainly involves S100 proteins, cathelicidin, and β-defensins. Cathelicidin is a synergistic agent for Th17 differentiation, and mice lacking cathelicidin are unable to produce adequate IL-17 levels in response to inflammation (155). β-defensins are key components of innate immunity that directly kill or inhibit the growth of pathogens (156). S-100 proteins can exert direct antifungal effects on fungal growth and indirect antifungal activity by regulating host immune responses (157). Previous studies have demonstrated that the expression of AMPs is increased in dermatophytosis (158) and that the reduced production of AMPs is related to chronic and widespread infection (159). Interestingly, some cytokines, such as IL-22 and TNF-α, synergize with IL-17 function by increasing AMPs production, thus playing an indispensable role in limiting the dissemination of pathogens (160, 161).

Several *in vivo* and *in vitro* studies have indicated the protective function of the Th17 immune response against dermatophyte infection. In guinea pigs infected with *T. benhamiae* and *A. vanbreuseghemii*, the *in situ* cytokine profile was characterized by the overexpression of transforming growth factor-β (TGF-β), IL-1β and IL-6 during infection, illustrating Th17 pathway involvement in the establishment of immunity (138). Increased levels of Th17 cells were also found in mice infected with *T. mentagrophytes* (162). Moreover, *Il-17ra* ^-/-^ or *Il17a/f* ^-/-^ mice suffered from a higher fungal load after inoculation of *M. canis* on the skin, which proved the role of the type 17 response in reducing the fungal burden (148). Consistent with this finding, patients with *STAT3* mutations have enhanced dermatophytosis and candidiasis susceptibility because of a diminished type 17 response (163). In addition, a reduction in type 17 immune activity has been linked to dermatophytosis (invasive or chronic) susceptibility, as has been indicated in adult T-cell leukemia/lymphoma (ATLL) individuals (164), those with *STAT3*-related malfunction (163), patients with gain-of-function (GOF) mutations (165), and those receiving anti-IL-17 antibody-related therapy (i.e., secukinumab and ixekizumab) (166–168). Thus, the type 17 immune response is essential for protecting the host from dermatophyte invasion.

The function of IFN-γ-modulated (Th1 or type 1) activities related to anti-dermatophyte-relevant skin immunoprotection is not adequately understood compared to IL-17-induced immune activities. Baltazar et al. (169) established an *Ifn-γ* ^-/-^ mouse model of *T. rubrum* infection and found that the fungal load increased significantly compared with that of WT mice. Moreover, *Ifn-γ* ^-/-^ mouse macrophages were less effective at engulfing *T. rubrum* conidia and killing them by generating ROS and IL-1β (169). Sardana et al. (146) therefore concluded that the elimination of a fungal infection is mainly mediated by an IFN-γ-induced (type 1 or Th1) response, as Th1 cells can produce proinflammatory cytokines and stimulate phagocytes.

However, in an *M. canis* infection model, WT mice with dermatophyte infection did not exhibit an elevation of the number of antigen-related IFN-γ-secreting T cells in the lymph nodes draining the skin (148). However, mice in which IL-17 expression was downregulated exhibited an alternative pathway of Th1 activity, indicating the presence of IFN-γ-regulated compensation for controlling *M. canis* infection. Nevertheless, studies with IL-17RA KO mice revealed that IFN-γ neutralization enhanced the secretion of Th17 lineage cytokines (IL-22, IL-1β, IL-17, and IL-6) in the skin and markedly suppressed fungal growth (148). Thus, IFN-γ deregulation is a possibility when IL-17 signaling is absent and might cause superficial *M. canis* overgrowth by suppressing type 17-linked activities. Similarly, individuals with *STAT1* GOF mutations that induce IFN gene transcription and alleviate IL-17-regulated immunity have an enhanced chance of developing chronic dermatophytosis and mucocutaneous candidiasis (165, 170). Regarding this finding, an elevated STAT1 response to Th1 cytokines (IL-27 and IFN-γ) inhibited IL-17-producing T-cell differentiation (171, 172). Enhanced STAT1 phosphorylation activities caused by IFN-γ can be inhibited after Janus kinase (JAK) inhibitor and ruxolitinib treatment (173). Interestingly, individuals with *STAT1* GOF mutations who are managed with ruxolitinib suffer from mucocutaneous candidiasis remission (174). The *M. canis* model experimental results are supported by the clinical data, indicating that the cause of dermatophyte susceptibility is correlated with IL-17-induced immune-related deficiency and that type 1 and 17 immunities oppositely modulate each other. The impact of a dysregulated Th1 response on dermatophyte infection in the absence of a functional type 17 immune response needs to be addressed in the future (175).

Nevertheless, in mice infected with *T. benhamiae*, the type 1 and type 17 responses were both found to be involved in fungal clearance, and persistent superficial infection occurred only when the IL-17 and IFN-γ pathways were both defective (176), suggesting that Th1 and Th17 responses function in a complementary manner. The contrasting results regarding the type 1 response in *T. rubrum*, *M. canis*, and *T. benhamiae* infection reminded us that different pathogens might have distinct virulence factors or activate unique subsets of APCs, thus selectively inducing the activation of different immune pathways. Briefly, the Th1 response may impair the protection mediated by the Th17 response but may also complement with the Th17 response to clear dermatophyte infection.

Treg cells ensure a controlled immune response in the presence of microbes, thereby preventing pathological immune responses (145). However, pathogen clearance might be hindered, and persistent infection can be promoted if Tregs excessively inhibit the immune response (146). Thus, maintaining a balance between immunological disease prevention and protective immune responses against pathogens requires optimal Treg activity and activation. Treg cell marker expression on peripheral blood CD4+ T cells was found to be considerably higher in individuals with persistent dermatophytosis than in healthy controls (177). Similarly, Kaya et al. (178) discovered that onychomycosis patients had a greater expression of CD25+ CD4+ Treg cells than healthy controls. These findings demonstrated that elevated Treg cell levels might contribute to vulnerability to dermatophytosis and infection persistence.

## 4 CARD9 deficiencies in deep dermatophytosis

Invasions of dermatophytes into the deeper tissues and even the hypodermis and dermis define the severe, resistant, and, in rare cases, fatal illness known as deep dermatophytosis. Deep dermatophytosis is more common in those who have had a solid organ transplant, are using immunosuppressants topically, or have a *CARD9* mutation (6).

CARD9 is a caspase recruitment domain-containing signaling protein essential for CLR downstream signaling and cross-signaling with other innate receptors (179). In addition, it is also involved in stimulating T-cell differentiation into different Th cells, initiating optimal adaptive immune responses (146). Therefore, CARD9 is vital for antifungal adaptive and innate immunity.

Patients with *CARD9* deficiency have impaired cytokine and chemokine production in response to fungal infections (180). A homozygous premature stop codon mutation (Q289) could be detected in 15 individuals within seven unrelated Tunisian and Algerian families, and a homozygous missense mutation (R101C) could be detected in two Moroccan related families, providing the first evidence linking autosomal recessive (AR) CARD9 deficiency and *T. violaceum-* or *T. rubrum*-mediated deep dermatophytosis (181). Subsequently, a CARD9 Q289 mutation was documented in Egyptian individuals suffering from *T. rubrum* affecting the skin, nails, and other superficial tissues (182), as well as in an Algerian women with *T. rubrum* infection of her brain (183). Since then, novel AR *CARD9* mutations, such as R101L and R70W, have been found in patients with deep and chronic dermatophytosis (184, 185). We summarized the *CARD9* mutations found in dermatophyte infection patients (**Table 3**) (181–186), more than 50% of whom are from North Africa. Interestingly, *CARD9* mutations are more common in Asia (187), however, African patients accounted for the majority of *CARD9* mutation-related dermatophytosis cases. One possible explanation is that different *CARD9* mutations causing different infections might have different geographic distributions. For example, homozygous (HMZ) p.Q289X, HMZ p.Q295X and HMZ p.D274fsX60 are the most commonly identified *CARD9* mutations, among which the HMZ p.Q289X mutation is mainly found in Africa and is associated with a significant increase in the risk of developing deep dermatophytosis compared to other mutations (187). This could explain the high prevalence of dermatophyte infection in African CARD9-deficient patients, as mutations are specific in particular populations or geographic regions.

**Table 3:** Summary of *CARD9* mutations in patients with dermatophyte infection.

Number	Country	Age of onset	Infection site	Consangui-neous marriage	Pathogen	Mutation	Deficiency of cytokine secretion	Th17 cells in PB	Treatment
Pedigree 1-1	Algeria	6	Skin, scalp, nails, LN	Yes	T. violaceum	p. Q289X	IL-6	Decrease	NR
Pedigree 1-2	Algeria	2	Skin, scalp, nails, LN, brain	Yes	*T. violaceum*	NR	NR	NR	NR
Pedigree 2-1	Algeria	9	Skin, scalp, nails, LN	Yes	*T. rubrum*	p. Q289X	IL-6	Decrease	NR
Pedigree 3-1	Algeria	8	Skin, scalp, nails	Yes	*T. violaceum*	p. Q289X	IL-6	Decrease	NR
Pedigree 3-2	Algeria	8	Skin, scalp, nails, LN	Yes	*T. violaceum*	NR	NR	NR	NR
Pedigree 3-3	Algeria	8	Nails	Yes	*T. violaceum*	p. Q289X	IL-6	Decrease	NR
Pedigree 4-1	Algeria	19	Skin, scalp, nails, LN	Yes	NR	p. Q289X	IL-6	Decrease	NR
Pedigree 4-2	Algeria	21	Skin, scalp, LN	Yes	NR	p. Q289X	IL-6	Decrease	NR
Pedigree 4-3	Algeria	NR	Skin, scalp	Yes	NR	NR	NR	NR	NR
Pedigree 5-1	Algeria	NR	Skin, scalp, LN	Yes	*T. violaceum*	p. Q289X	IL-6	Decrease	NR
Pedigree 5-2	Algeria	NR	Nails	Yes	NR	p. Q289X	IL-6	Decrease	NR
Pedigree 6-1	Morocco	NR	Skin, nails, LN, skeleton	Yes	*T. rubrum*	p. R101C	IL-6	Decrease	NR
Pedigree 6-2	Morocco	NR	Scalp, nails	Yes	NR	p. R101C	IL-6	Decrease	NR
Pedigree 7-1	Tunisia	6	Skin, scalp, nails	Yes	NR	p. Q289X	IL-6	Decrease	NR
Pedigree 7-2	Tunisia	12	Scalp, nails	Yes	*T. rubrum*	p. Q289X	IL-6	Decrease	NR
Pedigree 7-3	Tunisia	5	Skin, scalp, nails, LN	Yes	*T. rubrum, T. violaceum*	p. Q289X	IL-6	Decrease	NR
Pedigree 8-1	Tunisia	6	Skin, scalp, nails, LN	No	*T. rubrum, T. violaceum*	p. Q289X	IL-6	Decrease	NR
Sporadic 9-1	Egypt	13	Skin, nails	No	*T. rubrum*	p. Q289X	NR	NR	Posaconazole
Sporadic 10-1	Brazil	11	Skin	No	*T. mentagrophytes*	p. R101L	NR	NR	Itraconazole, KCZ, posaconazole, terbinafine, AmB
Sporadic 11-1	Algeria	47	Skin, scalp, nails, LN, brain	NR	*T. rubrum*	p. Q289X	NR	NR	Itraconazole
Pedigree 12-1	Turkey	8	Skin, oral cavity, nails, LN	Yes	*T. rubrum, T. violaceum, T. verrucosum*	p. R70W	IL-6	Decrease	Itraconazole, KCZ, terbinafine, FCZ
Pedigree 12-2	Turkey	NR	NR	NR	NR	NR	NR	NR	NR
Sporadic 13-1	USA	16	Skin, nails	NR	*T. rubrum*, *T. violaceum*, *Aspergillus fumigatus*, and *A. flavus*	p.Y91H	NR	Decrease	Griseofulvin, KCZ, itraconazole, posaconazole, AmB

AmB, amphotericin B; FCZ, fluconazole; IL, interleukin; KCZ, ketoconazole; LN, lymph node; NR, not reported; PB, peripheral blood.

*CARD9* deficiency is also related to an impaired type 17 immune response, since the Th17 cell proportion and IL-17A and IL-22 expression in *CARD9-*defective patients are significantly lower than that in healthy people (188). Interestingly, in individuals with both *STAT3* and *CARD9* mutations, high eosinophil and serum immunoglobulin E (IgE) levels were observed in addition to an increased susceptibility to fungal infections (189), which indicated an efficient biological mechanism that resulted in reduced Th17 pathway activity and enhanced Th2 pathway activity. Therefore, serum IgE and eosinophil levels could be utilized as indices in gene mutation tests for individuals with invasive dermatophytosis. In summary, the differentiation of Th cells (i.e., Th17) and the function of adaptive immune pathways were significantly impaired in *CARD9* mutant patients due to a deficiency in the production of proinflammatory cytokines (i.e., IL-6, IL-1β, TNF-α), resulting in increased susceptibility to chronic or deep fungal infections.

## 5 Conclusion

Dermatophytes are the most common pathogenic fungi worldwide, causing superficial and even deep infections. In the past few decades, scientists have made tremendous breakthroughs in understanding the pathogenicity of dermatophytes and the host immune responses against pathogenic fungi through *in vivo* and *in vitro* studies. With the increasing incidence of dermatophytosis and the emergence of drug-resistant *T. indotineae* strains (190), there is an urgent need for a better understanding of the virulence factors of dermatophytes and pathogen−host interactions to identify new targets for clinical treatment.

## Author contributions

RD: Conceptualization, Writing – original draft. XW: Writing – original draft. RL: Supervision, Writing – review & editing.

## Glossary

**Table:**

ATP-binding cassette transporter	ABC
Adult T-cell leukemia/lymphoma	ATLL
Antigen-presenting cells	APCs
Antimicrobial peptides	AMPs
Autosomal recessive	AR
B-cell lymphoma/leukemia 10	Bcl10
Caspase-associated recruitment domain 9	CARD9
C-type lectin receptors	CLRs
Cysteine dioxygenase	Cdol
Damage-associated molecular patterns	DAMPs
Dendritic cells	DCs
Dipeptidyl peptidase	Dpp
Family of serine hydrolases	FSH
Fc receptor γ-chain	FcγR
Gain-of-function	GOF
Immunoglobulin E	IgE
Immunoreceptor tyrosine-based activation motif	ITAM
Interferon γ	IFN-γ
Interleukin	IL
Janus kinase	JAK
Leucine aminopeptidase	Lap
Mannose receptor	MR
Metalloproteinases	Mep
Mucosa-associated lymphoid tissue lymphoma translocation protein 1	MALT1
Myeloid differentiation primary response 88	MyD88
Natural killer cells	NK cells
NOD-like receptor thermal protein domain-associated protein 3	NLRP3
Nuclear factor κB	NF-κB
Nucleotide-binding and oligomerization domain	NOD)-like receptors, NLRs
Pathogen-associated molecular patterns	PAMPs
Pattern recognition receptors	PRRs
Phospho-inositide-3 kinase	PI3K
Reactive oxygen species	ROS
Retinoic acid inducible gene	RIG)-like receptors, RLRs
Retinoic-acid-receptor-related orphan nuclear receptor gamma	RORγt
Squalene epoxidase	SQLE
Signal transducer and transcription 3	STAT3
Subtilisin-like serine protease	Sub
Sulfite efflux pump	Ssu1
T helper cells	Th cells
Toll IL-1 receptor domain	TIR)-comprising adaptor inducer interferon-β, TRIF
Toll-like receptors	TLRs
Transforming growth factor-β	TGF-β
Tumor necrosis factor α	TNF-α
Vascular endothelial growth factor	VEGF.
